# Supplementary material for: Non-communicable disease and mental health care during the COVID-19 pandemic in South Africa: Perspectives from selected healthcare professionals and patients
Source: PLoS One. 2025 May 5;20(5):e0318156. doi: 10.1371/journal.pone.0318156 (PMC12052180; doi:10.1371/journal.pone.0318156)
Supplement: Supplementary Fig 1 — . (DOCX) [file pone.0318156.s002.docx]

Other healthcare workers: Nurses, Pharmacists, Dieticians, Occupational Therapists, Optometrists and Physiotherapist

**Supplementary Figure 1: Characteristics of healthcare professionals (n=31)**
