# Supplementary material for: Non-communicable disease and mental health care during the COVID-19 pandemic in South Africa: Perspectives from selected healthcare professionals and patients
Source: PLoS One. 2025 May 5;20(5):e0318156. doi: 10.1371/journal.pone.0318156 (PMC12052180; doi:10.1371/journal.pone.0318156)
Supplement: Supplementary Fig 2 — . (DOCX) [file pone.0318156.s003.docx]

**Supplementary Figure 2: The degree to which healthcare professionals were negatively affected by the health system's inability to continue caring for patients with NCDs (n=21)**
