# Supplementary material for: Non-communicable disease and mental health care during the COVID-19 pandemic in South Africa: Perspectives from selected healthcare professionals and patients
Source: PLoS One. 2025 May 5;20(5):e0318156. doi: 10.1371/journal.pone.0318156 (PMC12052180; doi:10.1371/journal.pone.0318156)
Supplement: Attachment — Submitted filename: Response to reveiwer comments - 250924.docx [file pone.0318156.s008.docx]

PONE-D-23-32125

Non-communicable disease and mental health care during the COVID-19 pandemic in South Africa: perspectives from healthcare professionals and patients

Dear Editor

We are pleased to resubmit our revised manuscript. We thank the reviewer for his/her comments. We hope that the paper will now be acceptable for publication in PLOS ONE.

Please see below for the detailed responses to the Reviewer’s and Editor’s comments. The revisions have been included in the manuscript in the track and change format.

Yours sincerely

Prof Peer and colleagues

Thank you for revising your manuscript. Whilst the majority of the concerns have been addressed, per Reviewer 2's assessment, please do take care to ensure that neither your title nor your conclusions overstate the generalizability of your study. In addition, to ensure that your manuscript complies with our requirements for reproducibility and data availability, I would be grateful if you could please address the following points:

- Are you able to provide any additional details about the development of the questionnaires, including any pilot testing?

We have included the following text on Page 6: “The study questionnaire was developed through consultation with the COVID-19 ELEPHANT study group. The questions were tested for acceptability by two independent groups with diverse professional backgrounds, which further informed the development of the questionnaire.”

- Please provide a copy of the questionnaires as a supporting information file

The study questionnaire has been included.

- I noted a few references to data not shown. Can the specific data that support these statements be provided as a supplement?

We have included the data not shown previously in Supplementary Figures 1-4. We trust this is satisfactory.

Reviewer #2: Due to the small sample size and the use of private sector opinions in this study, it is suggested to modify the title of the article. The authors have corrected all the problems suggested by the first reviewer and have included them in the limitations of the study, but this study cannot be generalized to the whole country and it is better to include phrases such as "in the private sector" or "in a number of clinics" in the title of the article.

We have amended the title to include the word ‘selected’ and it now reads as follows: “Non-communicable disease and mental health care during the COVID-19 pandemic in South Africa: perspectives from **selected** healthcare professionals and patients”.

We could not include the suggestions made by the reviewer because the healthcare professionals were from both the public (n=13) and private (n=18) sectors. We could not include “clinics” because many health professionals worked in hospitals. We trust this is acceptable.
